# Rising Trends in Prostate Cancer Among Asian Men: Global Concerns and Diagnostic Solutions

**DOI:** 10.3390/cancers17061013

**Published:** 2025-03-17

**Authors:** Li-Chuan Ko, Nick Gravina, Joos Berghausen, Joe Abdo

**Affiliations:** 1 Department of Biochemistry, Molecular & Cellular Biology, Georgetown University Medical Center, 3900 Reservoir Rd NW, Washington, DC 20057, USA; lk864@georgetown.edu; 2 AstraZeneca, 1 Medimmune Way, Gaithersburg, MD 20878, USA; 3 Department of Pharmacology and Physiology, Georgetown University Medical Center, 3900 Reservoir Rd NW, Washington, DC 20057, USA; jhb109@georgetown.edu; 4 Oxford BioDynamics Inc., 9801 Washingtonian Blvd, Gaithersburg, MD 20878, USA

**Keywords:** prostate cancer, cancer epidemiology, Asian men’s health, global cancer trends, early detection, precision oncology, molecular diagnostics

## Abstract

Asia comprises 60% of the global male population, yet Asian men’s health issues, such as prostate cancer, remain sparse in the literature. Clinical researchers have often overlooked prostate cancer in Asian men, as available statistics suggest that Asian men are less likely to develop prostate cancer compared to Caucasian, Black, and Hispanic males. However, cultural investigations have indicated these figures may be influenced by factors such as cultural taboos, superstition and misconception, limited access to diagnostic tools and healthcare facilities, and epidemiological factors in terms of lifestyle, environmental exposure, and genetics. In particular, the lack of diagnostic resources, coupled with cultural stigmas surrounding cancers affecting the genitalia in Asian societies, may contribute to significant underreporting. Rapid aging population growth in East Asia and the continuing shift in Asiatic lifestyles towards Westernization will likely contribute to a continued rise in prostate cancer incidence among Asian men in the coming years. A comprehensive review of novel and commercially available prostate cancer detection tests demonstrates that accurate, less-invasive tools could relieve many of the pressures surrounding Asian men and their prostate health.

## Simple Summary

The majority of the global male population resides in Asia. Prostate cancer is rising rapidly across the entire continent due to factors such as aging populations, urbanization, and changing lifestyles. Cultural attitudes and taboos surrounding prostate health often delay early detection in many Asian countries. Traditional screening methods, such as PSA testing, yield high false positive rates and are not widely used across these healthcare systems. To address the rising burden of prostate cancer in Asia, there is a need for greater awareness, early screening, and improved urological health infrastructure. New diagnostic tools include the EpiSwitch Prostate Cancer Detection (PSE) test, which is a highly accurate blood test that has been clinically effective in detecting prostate cancer earlier and with greater accuracy. Its non-invasive nature helps overcome cultural barriers and preserves the dignity of Asian men, making it a vital advancement in prostate cancer diagnostics.

## 1. Introduction

Prostate cancer is a significant global concern for all men on Earth, as it is the second most diagnosed cancer and the fifth leading cause of cancer-related deaths among men globally [1,2]. According to the International Agency for Research on Cancer (IARC), there were 1,276,106 new cases of prostate cancer recorded in 2018 globally, accounting for 7.1% of all cancers in men [2]. This trend is expected to escalate, with incidence rates projected to increase by 79.7% worldwide by 2040 [2,3].

Despite its wide prevalence, the etiology of prostate cancer remains less understood compared to other cancers. While well-established risk factors include advanced age, ethnicity, genetic predisposition, and family history, additional contributing factors such as diet, obesity, chronic inflammation, hyperglycemia, infections, and environmental exposure to chemicals or ionizing radiation have also been identified [2]. Prostate cancer is the most diagnosed malignancy among elderly men, underscoring the role of age as a key risk factor [2].

**Figure 1:**
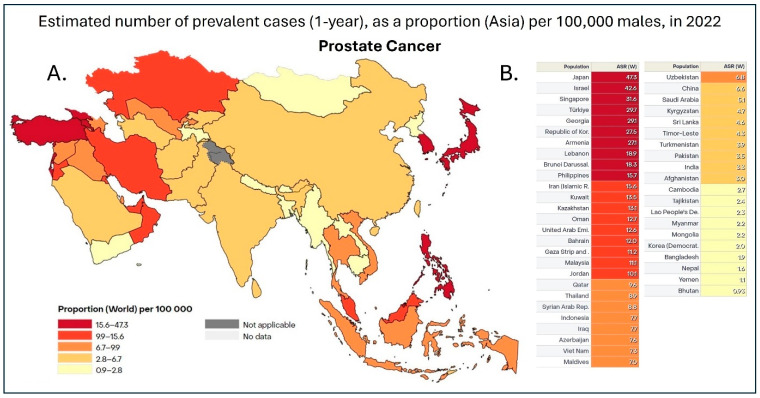
(**A**). Heatmap of 1-year prostate cancer prevalence in Asia in 2022. Data is shown as the age-standardized rate (ASR) per 100,000, with dark red representing countries with 15.6–47.3 cases, red representing 9.9–15.6 cases, dark orange being 6.7–9.9 cases, orange being 2.8–6.7 cases, and, finally, yellow representing 0.9–2.8 cases per 100,000 [7]. (**B**). Table with individual ASR based on individual countries in Asia. This dataset did not allow for the distinction between China and Taiwan; therefore, the data was pooled together [7].

The likelihood of developing prostate cancer varies significantly across ethnic groups. Statistics indicate that Black men are 64% more likely to develop the disease compared to Caucasian men [4]. Following this, Caucasian and Hispanic men have higher risk levels, while Asian men are statistically the least likely to develop prostate cancer [4,5]. However, cultural factors may obscure these statistics. Studies suggest that cultural sensitivities and taboos within Asian societies, particularly when cancer involves the genital regions, may lead to underreporting, fragmented data, and delayed diagnoses, ultimately affecting the perceived prevalence (Figure 1) in these populations. The Prostate-Specific Antigen (PSA) test is currently the gold standard for prostate cancer screening, which was developed in the 1970s and holds an accuracy rate of around 55% [6].

Rising prostate cancer rates among Asian men highlight a growing health challenge, yet cultural stigma around invasive screenings often hinders early detection. Innovative tests discussed in this comprehensive review offer less invasive alternatives to traditional methods, improving diagnostic accessibility while addressing privacy concerns and cultural hesitancy. These molecular diagnostic advancements not only aid timely intervention but also encourage proactive engagement regarding prostate health in communities where stigma and limited prostate health education persist.

**Figure 2:**
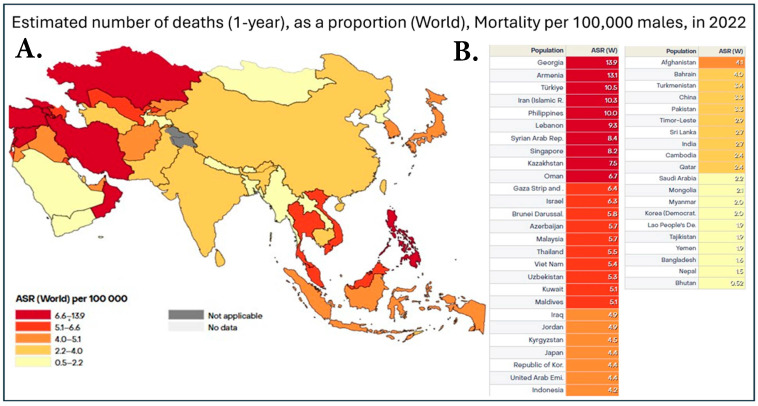
(**A**). Heatmap of prostate cancer mortality in Asia in 2022. Data is shown as the age-standardized rate (ASR) per 100,000, with dark red representing countries with 6.7–13.9 deaths, red representing 5.1–6.4 deaths, dark orange being 4.1–4.9 deaths, orange being 2.4–4.0 deaths, and, finally, yellow representing 0.52–2.2 deaths per 100,000. (**B**). Table with individual ASR based on individual countries in Asia. This dataset did not allow for the distinction between China and Taiwan; therefore, the data was pooled together [7].

Figure 1 illustrates a heatmap of the prevalence of prostate cancer in Asian countries, while Figure 2 shows a heatmap of prostate cancer mortality in the same Asian countries. When a country has both high cancer prevalence and mortality rates, accurately tracking these biostatistics is essential. For example, Japan has established a comprehensive cancer registry system, allowing for precise calculations of incidence and mortality rates [8]. This system not only enables meaningful comparisons between Japan’s cancer data and that of other countries, such as the United States, but also provides valuable insights into whether improvements in medical care and early detection are effectively addressing rising cancer rates. By examining these trends, we can assess whether healthcare advancements are leading to better outcomes or if increasing incidence continues to outpace improvements in treatment and prevention.

## 2. Cultural Taboos

In South Asia, cancer is often viewed as a stigma, perceived by some as a consequence of karma or a reflection of one’s sins, which can lead to individuals being blamed for their illness [9]. While the situation varies across regions, some countries where prostate cancer might be considered a taboo or sensitive issue include India, Pakistan, China, Sri Lanka, and Bangladesh. In these countries, cultural factors such as the importance of family honor, social stigma around cancer, and a general reluctance to discuss personal health challenges contribute to making prostate cancer a taboo subject. Discussing cancer, particularly when it affects the genitalia, is considered shameful by some, and such topics are often avoided in public educational discourse [9]. Studies by Shakil et al. [10] on prostate cancer awareness in South Asian countries further highlight this issue, revealing a significant lack of reliable knowledge regarding prostate cancer among South Asian men. Interestingly, South Asian women were found to have a better understanding of prostate cancer than men [10]. When Shakil’s [10] team questioned participants about prostate screening tests, the responses underscored a widespread lack of awareness and emphasized the need to end the taboo.

Additionally, 66% of male participants expressed discomfort and embarrassment at the thought of undergoing a Digital Rectal Examination (DRE), a procedure where a medical doctor examines a patient’s prostate gland for abnormalities by inserting their index finger into the patient’s rectum [11]. This further illustrates the impact of cultural taboos on prostate cancer detection and prevention efforts [10]. Miseducation and a lack of understanding might lead to underreporting, delayed diagnosis, and less public education on the subject, contributing to the perception that prostate cancer is less prevalent in this region of the world.

Likewise, studies by Pan et al. [12] on East Asian prostate cancer patients report a perceived loss of masculinity due to erectile dysfunction. Moreover, elderly Chinese men are often highly conservative regarding emotional expression, particularly on health issues, which discourages them from seeking medical help [12]. In Chinese culture, masculinity is idealized as strong, resilient, courageous, and “willing to shed blood rather than tears” [13]. This cultural view shapes men’s reluctance to express emotions or seek help for personal matters, especially health-related concerns.

Broad statistics show that Chinese prostate cancer patients often experience feelings of hopelessness, shame, and embarrassment, which can hinder help-seeking behaviors and lead to treatment delays [14]. Similar to the investigation conducted on the Chinese cohort and South Asian populations [15,16], prostate cancer patients often avoid seeking medical attention, viewing the disease as taboo and fearing treatment complications and side effects [17]. Malaysian healthcare professionals also note that patients often undergo the initial stages of grief, including denial and questioning of the veracity of the diagnosis [17]. This way of thinking, which views cancer as taboo and stigmatizes patients suffering from it, is not exclusive to Asian countries but is deeply rooted across Asian cultures [15,16]. These beliefs have permeated Asian culture, leading many to delay seeking medical attention until the pain becomes unbearable, resulting in late detection when the untreated disease has had time to metastasize if aggressive, ultimately increasing mortality rates [14,18,19].

In China, over two-thirds of patients are already diagnosed with advanced-stage disease at their initial visit [19], compared to the USA, where 70% of prostate cancer cases were diagnosed at an early, localized stage [20]. Patients presenting with advanced cancer not only contribute to the underreporting of cases but also endanger individual health and shorten survivability.

## 3. Superstition and Misconception

Superstition is a major contributor to delayed cancer diagnoses in Asia. In East Asian countries such as Japan, South Korea, Taiwan, and Mongolia, societal attitudes, cultural taboos surrounding men’s health, and the stigma associated with cancer often result in delayed diagnoses and lower rates of screening for prostate cancer. Patients in some of these regions often seek comfort and emotional support through religious practices, such as Taoism, believing that such practices can heal illness and relieve pain [3]. However, while religion may provide emotional support, there is no scientific evidence that religious practices improve physical health. The reliance on superstition can delay diagnosis and lead to disease progression and worse clinical outcomes. Similar trends have been observed in the Middle East, where individuals rely on religious beliefs to cope with illness, impacting medical decisions in a negative way [21].

Misconceptions also play a role in delayed detection. For instance, many believe that only the elderly (aged 65 and above) can develop prostate cancer, despite the increasing occurrence rate among men in their 50s and 40s. Also, those without a family history or lack of knowledge of prostate cancer among relatives often believe they are not at risk and may delay clinical action if symptoms arise [22,23,24].

Another widespread misconception is that prostate cancer screening always starts with a DRE. In fact, 60% of American men mistakenly believe an invasive procedure is the initial step in prostate health screening [25]. Even in Western cultures, where healthcare knowledge is more widespread, such misunderstandings about prostate cancer screening persist. In more conservative Asian communities, the belief that screening involves invasive, intimate procedures is even more likely to deter individuals from routine exams. Such trends are demonstrated in Shakil et al.’s research [10], showing that participants tend to avoid the topic of DRE, displaying a clear unwillingness to discuss it further, which ultimately leads to fewer of these prognostic procedures being performed.

## 4. Limited Access to Diagnostic Tools and Healthcare Facilities

The low incidence rate of prostate cancer in Asia does not accurately reflect the true prevalence of the disease, partly due to a lack of systematic PSA screening [26,27]. While incidence (Figure 1) and mortality rates (Figure 2) have been trending upward in recent years in countries such as Japan, Singapore, and China, PSA testing was rarely used in these regions before 2009 [19,28]. Increased adoption of PSA testing since then has corresponded with rising prostate cancer incidence in countries including Japan and Taiwan [29]. In developing Southeast Asian nations, such as the Philippines, Malaysia, and Thailand, the mortality-to-incidence ratio (MIR) (Table 1) is more than twice that observed in Japan and South Korea [29]. Financial constraints have historically been a significant factor in most Asian countries [1]. Many developing nations in Asia lack comprehensive healthcare insurance and well-equipped medical facilities to provide all stages of the patient journey after prostate cancer diagnosis [28,29].

In the past 5 to 10 years, the burden of prostate cancer has increased notably in economically diverse Southeast Asian countries, resulting in disparities in healthcare funding, insurance systems, and access to diagnostic, treatment, and innovative therapies [29]. Treatment guidelines and recommendations also vary by country, and limited public insurance coverage or additional financial resources for healthcare remain major barriers to accessing the most efficacious prostate cancer treatments approved in these regions [29]. For example, in China, large income disparities have led some families to forgo cancer screening due to financial limitations and lack of sufficient healthcare coverage [14].

In South Asian countries such as India, prostate cancer also appears underdiagnosed, with the limited number of healthcare institutions likely skewing the true incidence and mortality rates [1]. Patients in areas without PSA screening tend to present with more advanced disease than those in regions where PSA screening is available [29]. However, given current governmental healthcare spending, it is unlikely that PSA-based screening will be widely implemented across most Asian countries in the near future [1]. It should be noted that some Western countries, such as the United Kingdom, do not have a national screening program for prostate cancer either.

In Asia, particularly in East Asia, both healthcare professionals and the general public often view MRI and biopsy as the gold standard for prostate cancer screening [38]. However, the limitations of MRI for prostate cancer screening are often overlooked. The accuracy rate for MRI in detecting prostate cancer can be as low as 49%, with a specificity of 41% and a positive predictive value of 51% [6]. This indicates that MRI may lead to a high rate of false positives, where individuals are incorrectly identified as having the condition.

Prostate biopsies, on the other hand, carry risks of multiple adverse side effects, such as rectal bleeding (45%, American Cancer Society, Atlanta, GA, USA [39]), hematospermia (65.8%, Rosario et al. [40]), incontinence (6.7%, Harvard Medical School, Boston, MA, USA [41]), and erectile dysfunction (33%, Priority Men’s, Atlanta, GA, USA [42]). Furthermore, low-grade or indolent prostate cancers may be indistinguishable from benign prostatic hyperplasia (BPH) during analysis [43]. Non-visible lesions on an MRI, either from low-grade prostate cancer or tumors in hard-to-detect locations, could result in biopsies being taken from non-cancerous areas. This underscores the importance of incorporating a biomarker with high specificity for effective prostate cancer screening to mitigate the clinical limitations of the gold standards of prostate cancer detection [44].

## 5. Lifestyle, Environmental Exposure (Westernization), and Genetics

Asian populations exhibit significant diversity in diet and lifestyle, both of which play crucial roles in prostate cancer development and progression. In Western countries, diets are often rich in animal products and processed foods, while Eastern diets typically have fewer calories and include higher levels of essential nutrients [1,45]. Factors such as diets high in saturated fats and dairy products, low vegetable intake, excessive alcohol and tobacco consumption, and environmental forces also contribute to prostate cancer risk [28]. Generally, East and Southeast Asians consume more vegetables, less animal protein, and fewer high-fat foods than Western populations [1]. Research indicates that high consumption of red meat, fat, dairy products, and eggs correlates with an increased risk of prostate cancer [2,26]. Excessive consumption of red meat, which is rich in saturated fat and cholesterol, may also displace plant-based foods in the diet [26]. Studies indicate that the rising consumption of fat and red meat is linked to an increased risk of prostate cancer [2,26,45]. Dairy products, which may contain calcium, zinc, and other nutrients, have also been associated with an increased risk of prostate cancer [26]. Frequent consumption of fruits and vegetables, which are rich in vitamins, minerals, and plant-based compounds, is believed to lower cancer risk and mortality [2,26]. Green tea, a staple beverage in Asia, contains epigallocatechin-3-gallate (EGCG), an antioxidant that may protect against cancer [2,26,45].

Research by Kurahashi et al. [46] found that consuming green tea (3–4 cups per day or more) was associated with a reduced risk of advanced prostate cancer [26]. Similarly, Jian et al. [47] observed a lower risk of prostate cancer with higher green tea consumption [26]. Soy products, common in traditional Chinese and Japanese diets, are rich in isoflavones such as genistein [26]. This chemical compound has demonstrated anti-cancer properties by inhibiting the growth of prostate cancer cells, notably through counteracting the effects of the insulin-like growth factor I (IGF-I), which promotes cell proliferation [48]. Isoflavones are believed to protect against prostate cancer through their hormone-like properties [49]. By mimicking estrogen in the body, they bind to estrogen receptors, specifically estrogen receptor beta (ERβ) [50]. Activation of ERβ is thought to provide protective effects against prostate cancer by suppressing cell proliferation and inducing apoptosis [50]. Research conducted by Nagata et al. [51] provides further evidence that total isoflavones are significantly linked to a reduced risk of prostate cancer. Overall, soy consumption in Asian populations is linked to a 25–30% reduction in prostate cancer risk [45,52].

However, with the rapid economic growth in Asia, countries such as Japan, Korea, Taiwan, and China have experienced a significant rise in the consumption of animal fats over the past 40 years [1]. Korea and China have seen increases of 578.6% and 591.7%, respectively, in animal fat consumption between the 1960s and 2000s [1]. This dietary shift, known as nutrition transition, has contributed to the rising incidence of prostate cancers in these countries over recent decades [1]. While some studies found no direct association between alcohol consumption and prostate cancer risk, research by Tyagi et al. [53] in India and Hu et al. [54] in China identified a higher risk of prostate cancer linked to alcohol intake. Heavy alcohol consumption—defined as more than 15 g of ethanol per day or over three alcoholic drinks daily—has been suggested as a potential risk factor for prostate and other cancers [2].

Both active and passive exposure to cigarette smoke is widely acknowledged as a carcinogenic factor for many cancers, including prostate cancer. Smoking may increase prostate cancer risk through hormonal and genetic mechanisms [2]. Studies show male smokers often exhibit elevated levels of circulating sex hormones, which can promote cancer development and progression [2]. Genetic variations in the metabolism of polycyclic aromatic hydrocarbons (PAHs), carcinogenic compounds in cigarette smoke, may play a role in cancer onset and progression [2]. Studies have identified a dose-response relationship, where an increased number of cigarettes smoked per year correlates with higher prostate cancer mortality risk, particularly in cases diagnosed within 10 years of smoking [2]. While smoking is believed to elevate prostate cancer risk by altering steroid levels and introducing multiple carcinogens, findings on the relationship between smoking and prostate cancer risk remain inconsistent, with some studies reporting no significant association [55,56].

Prostate cancer incidence (Figure 1) displays notable geographic and environmental variations among Asian men [5,45]. Research reveals that immigration and relocation of Asian men to Western countries or countries with Western diets and lifestyles, such as the United States, United Kingdom, and Singapore, exhibit significantly higher rates of prostate cancer compared to those in their countries of origin [1,27,28]. For instance, prostate cancer incidence in Indian immigrants to these regions surpasses that of Indians residing in India [1]. Similarly, Chinese-American men face a three to five-fold higher risk of developing prostate cancer than Chinese men living in Asia [27,45,57]. Prostate cancer ranks among the three most frequently diagnosed cancers in 9 out of 10 Asian populations assessed in Hawaii and other U.S. regions, with Laotians being the sole exception [27].

Furthermore, in 2016 alone, Asian-American, Native Hawaiian, and Pacific Islander men accounted for an estimated 4550 new cases of prostate cancer, making up approximately 18% of all cancers diagnosed within these groups [28]. Research highlights that geographical and environmental factors significantly contribute to variations in prostate cancer incidence across populations with shared ethnic backgrounds. These patterns are heavily influenced by increased exposure to Western dietary habits and improved access to healthcare services, including more frequent PSA screening [5,27,52]. Baade et al. [58] suggested that differences in healthcare systems, screening availability, and lifestyle adjustments largely explain the higher prostate cancer rates among Asian immigrants compared to native Asians [28].

The significant rise in prostate cancer cases among Asian-American men underscores the impact of environmental factors such as Westernized diets and improved healthcare access [27,45,57]. Studies also conclude that geographic variations in prostate cancer incidence among the same ethnic groups highlight the critical role of environmental influences in disease risk [1,27,28]. The transition to Western lifestyles, combined with improved screening practices, contributes substantially to the increasing burden of prostate cancer in Asian-American and Asian populations [1,45].

Variations in genetic backgrounds between Asian and Western populations may also attribute to the significant differences in prostate cancer incidence. Chung et al. [26] collected genome-wide association studies in Asian populations and identified significant genetic markers associated with prostate cancer risk. Polymorphisms in genes related to cytochrome P450 and variations in the 8q24 region of chromosome 8 have been specifically linked to elevated prostate cancer risk [26]. Additionally, single nucleotide polymorphisms associated with prostate cancer in European populations have been replicated in Chinese, Malaysian, and Japanese men, highlighting shared genetic factors across ethnic groups [26].

The genetic diversity among Asian ethnic groups adds another layer of complexity to understanding these disparities. East and Southeast Asians largely share a common Han and Mongolian ancestry, while South Asians exhibit a mixed genetic background combining Caucasian and Mongolian traits [1]. In contrast, West Asians represent a blend of Caucasian and Arabian populations [1]. These perhaps explain the high incidence rate in Israel among most Asian populations [27]. The high incidence rates in Asia (Figure 1) are observed in countries such as Israel, Singapore, Japan, and the Philippines—regions often considered more developed and Westernized [27]. Moreover, countries with closely related cultures and origins, such as Japan and Korea or Iraq and Iran, tend to exhibit similar age-standardized incidence rates of prostate cancer [27]. This demonstrates the potential influence of lifestyle and environmental factors alongside genetic predispositions as impacting forces on increasing the incidence of prostate cancer in Asia.

## 6. Trends and Aging

The incidence of prostate cancer is rising rapidly in Asia (Figure 1) [5,26]. By 2040, an estimated 1,017,712 new cases of prostate cancer are expected worldwide, reflecting a 79.7% overall increase [2,28]. Asia alone is expected to experience a 100.9% increase, with the highest rises anticipated in Africa (+120.6%) and Latin America and the Caribbean (+101.1%) [2]. Prostate cancer has become a significant health concern across East and Southeast Asia, affecting both developed and developing nations [26,29,45].

Among 42 Asian countries with available data, prostate cancer ranks among the top three most common cancers in 16 of them [27]. In Japan, the incidence has surged sharply since the latter half of the 20th century, with one in five men now diagnosed with the disease [59]. By 2023, prostate cancer had become the most prevalent cancer among men in Japan [60]. Similarly, South Korea has witnessed a notable annual percentage increase of 12.8% [45], making prostate cancer the leading cancer in men in 2023 [61]. In Taiwan, prostate cancer moved from being the eighth most common cancer in men in 1988 to the fifth position by 2016, reflecting a significant upward trend [62]. In Singapore, prostate cancer became the most common cancer in men, accounting for 16.8% of all male cancer cases by 2021 [63]. In China, the incidence of prostate cancer is also rapidly growing, with an annual increase of 11.5% [19]. By 2020, approximately 115,000 new cases were reported, representing 4.7% of all male malignancies [19]. Developed regions such as Shanghai reported a high prostate cancer incidence rate of 8.3%, ranking it the fourth most common cancer in men by 2016 [64]. Similarly, Hong Kong has seen a doubling of prostate cancer cases over the past decade [22]. In 2017, there were 2240 new cases, a 17% increase from 2016, making it the third most common cancer among men [18,22]. By 2022, prostate cancer remained among the top three leading cancers in Hong Kong [65]. Prostate cancer is also a serious concern in Malaysia, where it accounted for 9.5% of all male cancer cases and ranked as the third most common cancer in 2022 [66]. In South Asia, particularly India, prostate cancer is the third leading cancer among men of all ages, accounting for 6.1% of cases [67].

Among men over 65, the incidence rate rises to 12.3%, making it the second most common cancer in older men [67]. Table 1 illustrates regional differences in healthcare indices and trends, reflecting the varying degrees of disease burden and healthcare infrastructure across countries. Countries such as Japan, South Korea, and Singapore have shown significant surges in prostate cancer incidence, while others, such as Malaysia and the Philippines, face steadily increasing trends. These observations are mirrored in the Middle East, where prostate cancer incidence rates also vary widely, influenced by ethnicity, healthcare access, and regional disparities.

In the Middle East, the prostate cancer incidence rate varies by country and ethnicity. It is ranked first in Cyprus (26.5%), Israeli Jews (22.5%), and SEER men (28.5%), second in Turkey’s Izmir region (13.7%), third among Israeli Arabs (11.6%), and fourth in Jordan (8.2%) [68]. These trends highlight the growing burden of prostate cancer across Asia and the Middle East. The increasing incidence underscores the need for improved awareness, early detection, and healthcare interventions to address this emerging public health challenge. While PSA testing is not widely used in many Asian countries, its increased adoption has contributed to higher reported incidence rates in developed countries [28]. Despite lower incidence rates compared to Western countries, the disease is becoming increasingly prevalent in East Asia, where 23.6% of the world’s male population resides and 8.2% are aged 65 or older [45]. This underscores the urgent need for enhanced awareness, screening, and healthcare infrastructure in the region.

Beyond genetics and family history, aging is also a well-established risk factor for prostate cancer [26]. In Asia, the incidence and mortality rates of prostate cancer (Table 1) are rising significantly due to an aging population, increasing urbanization, and the adoption of Westernized lifestyles [28]. As these demographic and lifestyle changes continue, prostate cancer is projected to become an even more pressing healthcare and socio-economic challenge in the region [1]. With ongoing population aging and the accelerated pace of lifestyle changes driven by economic development, the burden of prostate cancer is expected to grow rapidly [1]. Notably, 70% of prostate cancer-related deaths occur in patients aged 75 and older, as observed in both the United States and Japan [27]. Addressing this growing burden will require a concerted effort to implement region-specific early detection, prevention, and treatment strategies, ensuring equitable access to care for aging populations across Asia.

The mortality-to-incidence ratio (MIR) is commonly used to assess the effectiveness of a population’s cancer control program. The ratio signifies that a population’s mortality rate has been normalized to its incidence rate. Interestingly, for Asian countries with a higher incidence rate than the global average, such as Japan and South Korea, the MIR was much lower than the global average (Table 1). A similar trend was observed with the countries that had much lower incidence rates than the global average, with all demonstrating a much higher MIR compared to the global average (Table 1). This data shows that the incidence rate is directly correlated to MIR and, thus, the efficacy of the cancer control program, suggesting that countries with higher-than-average MIR should focus more on active surveillance in order to improve their cancer control programs.

## 7. New Diagnostic Tools for Prostate Cancer Detection

The PSA test is the current gold standard for evaluating prostate health and assessing disease status, as it is the most widely used technique globally [52]. The PSA test detects the prostate-specific antigen, a glycoprotein enzyme produced by both normal and cancerous prostate cells. Its levels in the blood can indicate abnormalities in the prostate. However, it is important to note that PSA testing does not diagnose prostate cancer directly. Instead, it serves as a screening tool to flag potential issues that require further clinical investigation [69].

PSA levels can rise due to various conditions, such as non-cancerous inflammation, acute prostatitis, and benign prostatic hyperplasia [69]. The difficulty in distinguishing these conditions from early-stage and late-stage prostate cancer often results in misdiagnosis, leading to high rates of false positives, which, in turn, prompts unnecessary medical treatment, including invasive transrectal or transperineal biopsies [69]. For example, when PSA levels range between 4 and 10 ng/mL, approximately 80% of the positive cases are not due to prostate cancer [59]. This demonstrates the test’s limitation in distinguishing between malignant and benign conditions of the prostate. Given that most current prostate cancer research has been conducted on Western populations [45], screening and treatment protocols must be tailored to the epidemiological and socioeconomic contexts of Asian countries [27]. Applying race-specific approaches to prostate cancer diagnostics and treatment is essential to improve outcomes in diverse populations [45].

Two of the most prominent commercially available prostate cancer tests are 4Kscore^®^ (Elmwood Park, NJ, USA) and ExoDx™ (ExosomeDX, Waltham, MA, USA). The 4Kscore^®^ test is a blood test that measures the levels of four prostate-specific antigens: Total PSA, Free PSA, Intact PSA, and Human Kallikrein-2 (hK2) [70]. It has demonstrated a positive predictive value (PPV) of 34%, a negative predictive value (NPV) of 96%, and a specificity of 27% (Table 2) [71,72]. While the 4Kscore^®^ test is more accurate than traditional PSA testing in evaluating the risk of aggressive prostate cancer, primarily because it is not influenced by benign prostate conditions, e.g., BPH [73], it does not directly diagnose prostate cancer. Its results require integration with other clinical findings, such as those from a DRE, MRI, or biopsy. However, the 4Kscore^®^ test was developed using data from a predominantly Caucasian demographic, with study participants comprising 49% Caucasian, 7% Black, 6% Asian, and 38% from other racial groups, including 9% non-disclosure [72]. This limited diversity raises concerns about the test’s generalizability and accuracy for underrepresented populations.

The ExoDx™ test, in contrast, is a urine-based assay that measures the expression levels of three prostate cancer-associated genes: PCA3, ERG, and SPDEF [74]. It has a PPV of 36%, an NPV of 91%, and a specificity of 34% (Table 2) [75]. Similar to the 4Kscore^®^ test, ExoDx™ provides a risk assessment rather than a definitive diagnosis and must be considered alongside other clinical evaluations. The test’s development was also conducted in a study population skewed heavily toward white participants, comprising 69% Caucasian, 22% Black, 3% Asian, 2% Hispanic, and 4% from other ethnic groups [76]. This racial imbalance further limits its applicability to non-Caucasian patients.

Several other commercially available prostate cancer detection tests are assessed in Table 2, where their accuracy, PPV, NPV, and specificity and sensitivity are compared. Many of these tests share the same limitations as traditional PSA testing, especially low specificity, which often leads to high false-positive rates [77]. This can result in increased patient anxiety, unnecessary biopsies, and ultimately higher healthcare costs [78], underscoring the need for further advancements in prostate cancer diagnostics to improve both specificity and inclusivity. As shown in Table 2, 4Kscore^®^, ExoDx™, and MyProstateScore 2.0 demonstrate high NPV, suggesting that these tests are effective at ruling out the disease in individuals with a negative result. However, these tests sacrifice or neglect the performance of their specificity, where a lower ability to rule out cancer increases false positives and unnecessary confirmatory procedures.

**Table 2:** Comprehensive comparison of prostate cancer diagnostic tests, focusing on their performance metrics, including accuracy, PPV, NPV, specificity, and sensitivity. Tests are listed in descending order of accuracy [6,71,72,75,79,80,81,82,83,84,85,86,87].

Test	Accuracy	PPV	NPV	Specificity	Sensitivity
Galleri	--	--	--	--	11%
EpiSwitch PSE	94%	93%	95%	97%	86%
PHI	63%	59%	88%	53%	90%
Stockholm3	59%	53%	84%	33%	92%
Select MDx	57%	42%	82%	50%	77%
IsoPSA	55%	69%	72%	42%	89%
PSA	55%	25%	86%	53%	64%
MyProstateScore 2.0	50%	28%	97%	32%	96%
ExoDx	50%	36%	91%	34%	92%
Proclarix	40%	47%	80%	19%	94%
4Kscore	38%	34%	96%	27%	97%

The EpiSwitch^®^ Prostate Screening (PSE) test is an innovative binary blood assay leveraging five epigenetic biomarkers that target chromatin conformation signatures [79,88]. Its clinical validation, involving a diverse cohort of 38% Caucasian, 32% Black, 23% Asian, and 7% other participants, enhances its applicability across varied populations [89]. To our knowledge, the PSE algorithm was trained with the highest percentage of Asian genomes compared to other commercially available tests for prostate cancer detection. By analyzing whole blood immune cells, the test examines the three-dimensional structure of non-coding DNA within the nucleus, which regulates gene expression [90]. These 3D genomic structures are preserved during cell division and carry vital regulatory and environmental information that reflects an individual’s phenotype [90]. This approach enables the identification of chromatin conformation signatures strongly associated with the presence and progression of prostate cancer [88]. By integrating these biomarkers with PSA data, the PSE test delivers high diagnostic accuracy and specificity, offering insight into both healthy and diseased states [88].

Research conducted by Pchejetski et al. [79] highlights both the limitations and potential of PSA testing in prostate cancer diagnostics. While PSA testing alone demonstrated a PPV of 14%, an NPV of 93%, and a specificity of 33%, the introduction of the EpiSwitch Prostate Screening (PSE) test represents a significant advancement [79]. The PSE test, which incorporates PSA as a continuous variable, achieves an impressive combined PPV of 93%, NPV of 95%, and specificity of 97% (Table 2) [79]. This innovation aligns with the National Comprehensive Cancer Network (NCCN) guidelines, which emphasize the importance of “considering biomarkers that improve the specificity of screening” [44]. Specificity evaluates a test’s ability to accurately identify individuals who do not have the disease, reducing the likelihood of false positives. This improves patient outcomes and reduces the need for unnecessary biopsies, along with their associated risks. The breakthrough of the EpiSwitch PSE represents a transformative step in prostate cancer diagnostics, providing a more reliable and precise method for detecting the disease.

## 8. Conclusions

Academic literature and prevailing data suggest that Asian men are less likely to develop prostate cancer compared to Black, Caucasian, and Hispanic populations—attributed to factors such as genetic predispositions, lifestyle choices, and dietary habits. The rising incidence of prostate cancer among Asian men highlights a pressing global health challenge shaped by cultural, socio-economic, and environmental influences. Historical underreporting, driven by cultural taboos, limited access to innovative diagnostic tools, and deeply rooted misconceptions have masked the true prevalence of the disease.

Commercially available tests that offer both high accuracy and specificity effectively reduce false positives, which, in turn, helps lower unnecessary healthcare costs. Non-invasive tests can also alleviate the cultural pressures faced by Asian men, as assessing prostate health via a blood draw can circumvent the need for less accurate and uncomfortable procedures such as rectal examinations, thus reducing stigma and taboos. Among the 11 commercially available tests today, EpiSwitch PSE stands out in terms of performance and ease of use due to small sample requirements and can be performed on any aged male with any PSA score. It is currently available for clinical use in both the United Kingdom and the United States. PSE is utilized by primary care providers, urologists, radiation oncologists, and concierge physicians as frontline and serial testing for prostate cancer detection. Importantly, it has already been used in thousands of patients in the United States. A key feature of EpiSwitch PSE is its detection stability, with a shelf life of up to 30 days at ambient temperature. It requires only 2–3 mL of blood without centrifugation or temperature controls, making it easy to deploy and access globally. Such tests would be especially valuable in developing countries and rural areas in Asia, where diagnostic tools and healthcare facilities are often limited.

As Westernization accelerates dietary and lifestyle forces on Asian men, coupled with aging populations, the risk of prostate cancer is projected to escalate significantly. This highlights an urgent need to address factors such as conservative attitudes toward cancers related to genitalia, inadequacies in healthcare systems, and the lack of advanced diagnostic tools. Promoting culturally sensitive awareness campaigns, implementing effective diagnostic technologies, and developing tailored healthcare policies are essential to mitigate the growing public health impact of prostate cancer and ensure equitable access to early detection and treatment for Asian men.

Implementing molecular diagnostic technologies could play a pivotal role in containing the impending prostate cancer pandemic in Asia. These innovations accurately differentiate clinically significant prostate cancer from benign conditions through a simple blood draw, encouraging more screenings that circumvent embarrassment and facilitating timely medical intervention for those truly at high risk. By ensuring equitable access to advanced diagnostic tools and encouraging advocacy and education to augment medical trust, we can enhance early detection and treatment strategies for Asian men, addressing a rising healthcare challenge with precision and efficiency.

## Figures and Tables

**Table 1:** Prostate cancer statistics for selected countries in the Asia-Pacific region, the US, and globally. It includes incidence rates, mortality rates, mortality-to-incidence ratio (MIR), annual new cases, and healthcare index rankings for 2024. Countries are listed in descending order of healthcare index scores, with trends in disease progression also highlighted. MIR assesses the burden of a disease and measures the efficacy of cancer control programs between regions [30,31,32,33,34,35,36,37].

Country	Incidence Rate per 100k	Trend	Mortality Rate per 100k	Mortality to Incidence (%)	New Cases per Year	Health Care Index 2024
Taiwan	31.9	Rising rapidly	10.5	32.9	7500	78.72^
South Korea	29.3	Rising rapidly	4.4	15.0	16,374	77.70
Japan	51.8	Rising	4.5	8.6	106,139	59.52
Singapore	32.8	Rising rapidly	8.2	25.0	2020	57.96
India	5.6	Rising	2.7	48.2	37,948	45.84
China	17.3	Steadily Rising	8.0	46.2	153,448	41.40
Thailand	12.5	Steadily Rising	5.5	44.0	7830	33.01
Philippines	24.1	Rising	10.0	41.5	9764	32.55
Malaysia	12.9	Steadily Rising	5.7	44.2	2360	32.52
USA	116.5	Steady	18.8	16.1	230,125	56.71
Global	29.4	Steadily Rising	7.3	24.8	1,467,854	--

^ Highest in the world.
